# Influence of the energy and digestible lysine contents of the diet on performance and egg quality traits of brown-egg laying hens from 19 to 59 weeks of age

**DOI:** 10.1016/j.psj.2021.101211

**Published:** 2021-04-21

**Authors:** R. Scappaticcio, J. García, G. Fondevila, A.F. de Juan, L. Cámara, G.G. Mateos

**Affiliations:** ⁎ Camar Agroalimentaria S.L., Toledo, Spain; † Departamento de Producción Agraria, Universidad Politécnica de Madrid, 28040 Madrid, Spain

**Keywords:** brown laying hens, digestible lysine requirements, egg production, egg weight, metabolizable energy

## Abstract

The influence of nutrient density and standardized ileal digestible lys (**DLys**) content of the diet on egg production and egg quality traits, was studied in brown-egg laying hens from 19 to 59 wk of age. The experimental design was completely randomized with eight treatments arranged as a 2×4 factorial with two AMEn concentrations (2,680 and 2,780 kcal/kg) and four levels of DLys (0.68, 0.72, 0.76, and 0.80%). Each treatment was replicated nine times and the experimental unit was a cage with nine hens. Hen production, egg components (proportion of albumen, yolk, and shell), egg quality traits (Haugh units, egg shell strength, and incidence of broken, dirty, and shell-less eggs) were measured by period (28 d) and cumulatively. Data were analyzed as a completely randomized design with energy concentration, level of DLys, and their interactions as main effects. In addition, the effects of the level of DLys on the variables studied, were partitioned into its linear and quadratic components. No interactions between AMEn and DLys content of the diet were detected for any of the traits studied and therefore, only main effects are presented. An increase in the AMEn concentration of the diet from 2,680 to 2,780 kcal/kg increased energy intake (*P* < 0.05) and egg weight (*P* < 0.001) and improved feed conversion ratio (*P* < 0.05). An Increase in DLys from 0.68 to 0.80% did not affect the number of eggs produced but increased linearly egg weight (*P* < 0.01) and egg mass production (*P* < 0.05). Diet did not affect egg quality. In conclusion, an increase in the AMEn content of the diet from 2,680 to 2,780 kcal/kg increased egg weight and improved feed efficiency. Laying hens require no more than 744 mg DLys/d (corresponding to 0.68% DLys) to optimize egg production. However, when the objective is to maximize egg weight, hens should consume at least 843 mg DLys/d (corresponding to 0.76% D Lys).

## INTRODUCTION

Apparent metabolizable energy and standardized ileal digestible lys (**DLys**) contents of the diet are two key variables affecting egg production and feeding cost in commercial laying hen operations. Usually, an increase in the energy content of the diet decreases proportionally feed intake (**FI**) because hens tend to adjust feed consumption to satisfy their energy requirements (Grobas et al., 1999a; Harms et al, 2000; Wu et al, 2005). However, when the diet is diluted excessively, hens might not be able to maintain FI to meet their needs for egg production, and BW gain (Nielsen, 2004; Bouvarel et al., 2011; Pérez-Bonilla et al., 2012b; dePersio et al., 2015). Highly concentrated energy diets, however, are costly and might reduce feed efficiency for egg production, because part of the energy ingested is directed to fat deposition and BW gain (Perez-Bonilla et al., 2012a). On the other hand, high energy diets usually contain more linoleic acid (**LNL**) and supplemental fat than low energy diets (Grobas et al., 2001; Safaa et al., 2008b; Herrera et al., 2018) resulting often in an increase in egg size (Harms and Waldroup, 1963; DeGroote, 1972; Grobas et al. 1999b; Wu et al., 2005).

The effect of the AMEn content of the diet on egg quality traits is a subject of debate. Junqueira et al. (2006) observed a decrease in shell quality but no changes in albumen height, when the AMEn of the diet increased from 2,850 to 2,950 kcal/kg. In contrast, a similar increase in energy did not affect shell quality in the report of Gunawardana et al. (2008). On the other hand, Wu et al. (2005) observed that an increase in yolk weight when the AMEn of the diet increased from 2,720 to 2,955 kcal/kg whereas no effects were reported by Grobas et al. (1999b), Gunawardana et al. (2008), and dePersio et al. (2015).

Usually, commercial diets for laying hens are formulated based on the ideal protein concept in which DLys, the second limiting amino acid (**AA**) in laying hen diets, is used as a reference (Baker, 1997; Bregendahl et al., 2008; Lohmann, 2018). Consequently, accurate estimation of DLys requirements is essential to maximize egg production (Spangler et al., 2018; FEDNA, 2019), optimize egg cost (Novak et al., 2004) and reduce nitrogen excretion (Rocha et al., 2009; Kumar et al., 2018). The Lys requirements of laying hens for optimal egg production have been determined in numerous experiments, with high variability among estimates (Faria et al., 2003; Lemme, 2009; Kumar et al., 2018). In fact, under commercial practices, the recommendation for digestible Lys in mg/d for brown-egg laying hens consuming 110 g of feed/d in the peak phase is 858 for Isa Brown (2017), 715 for Lohmann (2018), 800 for Hy-Line (2018), 792 for CVB (2018), 814 for FEDNA (2018), and 759 for H&N (2019), equivalent to 0.78, 0.65, 0.72, 0.73, 0.74, and 0.69% of DLys, respectively. Early research reported that total Lys requirements of laying hens (mg/d) to maximize egg production varied between 522 (Bray, 1969) and 710 (Nathanael and Sell, 1980). In fact, an intake of 690 mg Lys (equivalent to 593 mg DLys/d) was recommended by the NRC (1994) for brown-egg laying hens. More recently, the digestible Lys requirements for white laying hens have been estimated within a range of 540 mg/d (Schutte and Smink, 1998; Bregendahl, 2008) to 856 mg/d (Pastore et al., 2018). In fact, Lemme (2009), Klein (2013), and Van Krimpen et al. (2015) recommend 830, 726, and 855 mg/d (equivalent to 0.75, 0.66, and 0.78% DLys) to maximize egg production in hens consuming 110 g of feed/d. The reasons for the discrepancies among authors are not well documented but include differences in ambient temperature, management practices, genetic background, stage of the egg production, diet composition, methodological criteria (i.e., broken line vs. quadratic polynomial regression), target variable evaluated (i.e., egg rate vs. egg mass vs. egg weight vs. feed efficiency), and unit of Lys (total vs. fecal vs. ileal) used to estimate the requirements.

The information available on the influence of the DLys content of the diet on egg quality traits is limited. Spangler et al. (2018) reported a significant reduction in egg specific gravity and Haugh units (**HU**) but no effects on the proportion of egg components, when the DLys content of the diet increased from 0.52% to 0.75%. Kumar et al. (2018) observed a linear increase in the proportion of cracked eggs as the level of DLys of the diet increased from 0.50 to 0.85 DLys. However, Novak et al. (2004) reported than an increase in the Lys content of the diet did not affect the proportion of shell of the eggs but increased that of albumen and decreased that of yolk.

The objective of this research was to determine the effects of increasing the energy concentration of the diet from 2,680 to 2,780 kcal AMEn/kg and the content in DLys from 0.68% to 0.80%, on performance and egg quality traits of brown-egg laying hens from 19 to 59 wk of age.

## MATERIALS AND METHODS

### Husbandry, Diets, and Experiment Design

The procedures used in this research were approved by the Animal Ethics Committee of the Universidad Politécnica de Madrid and were in compliance with the Spanish Guidelines for the care and use of animals in research (Boletín Oficial Del Estado, 2013). In total, 648 Lohmann Brown Classic hens were selected at random from a commercial flock at 18 wk of age and housed in the second floor of a cage battery system within an environmentally controlled barn (110,000 birds). Hens were weighed individually and allotted in groups of nine, with similar mean BW, to 72 adjacent enriched cages (120 × 63 cm and 45 cm of height; Facco S.p.A., Padova, Italy). The cages were provided with an open trough feeder and two low pressure nipple drinkers. Barn temperature was recorded daily throughout the experiment, with a minimum of 22 ± 2°C per day in January and a maximum of 26 ± 2ºC in September (last month of the experiment). The lighting program consisted in an increase of the light period to reach 16 h at 21 wk of age and then maintaining the hours of light constant. Birds had free access to feed in mash form and water throughout the experiment. There were eight experimental diets arranged as a 2 × 4 factorial with two energy concentrations (2,680 vs. 2,780 kcal AMEn/kg) and four levels of DLys (0.68, 0.72, 0.76, and 0.80%). All the other indispensable AA were formulated according to the ideal protein concept (FEDNA 2018). To ensure that DLys was the AA limiting hen production in all cases, the diets were formulated to exceed, at least by 3 percent units, the desired indispensable AA (**IDAA**) to DLys ratio. As a consequence, compared to the ideal protein content recommended by FEDNA (2018), the calculated IDAA to DLys ratio of the experimental diets was 91 versus 88% for TSAA, 73 versus 70% for Thr, 24 versus 21% for Trp, 93 versus 89% for Val, 83 versus 80% for Ile, and 135 versus 104% for Arg. Prior to feed formulation, the three main ingredients used (corn, soybean meal, and sunflower meal) were analyzed for CP, CF, ash, EE, and AA to insure that the chemical and nutritional values were similar to those reported by the FEDNA (2019) feed composition tables. For the manufacturing of the experimental feeds, the two extreme diets of each set (high and low energy diets) were formulated. Within each of the two energy levels, the intermediate diets resulted from the mixing in adequate proportions of the two extreme diets. The ingredient composition and the calculated and determined nutrient content of the experimental diets are presented in Table 1.

**Table 1:** Ingredient composition and physico-chemical analyses of the experimental diets (%, as fed basis).

	2,680 kcal AMEn/kg				2,780 kcal AMEn/kg			
DLys^1^	0.68%	0.72%	0.76%	0.80%	0.68%	0.72%	0.76%	0.80%
Ingredient								
Corn	64.33	62.16	59.93	57.76	61.50	59.19	56.82	54.51
Soybean meal (47% CP)	14.86	16.91	19.02	21.07	15.71	17.81	19.98	22.08
Sunflower meal (36% CP)	9.55	9.35	9.15	8.96	8.67	8.55	8.42	8.30
Calcium carbonate^2^	8.34	8.34	8.34	8.34	8.96	8.96	8.96	8.96
Soy oil soapstocks	0.78	1.09	1.42	1.73	2.97	3.31	3.66	4.00
Dicalcium phosphate	1.27	1.26	1.25	1.24	1.34	1.32	1.30	1.28
Sodium chloride	0.36	0.36	0.36	0.36	0.35	0.35	0.35	0.35
L-Lysine-HCl (78%)	0.12	0.11	0.10	0.09	0.11	0.10	0.08	0.07
DL-Methionine (99%)	0.16	0.18	0.20	0.22	0.16	0.18	0.20	0.22
L-Threonine (98%)	0.02	0.02	0.02	0.02	0.02	0.02	0.02	0.02
L-Tryptophan (98%)	0.01	0.01	0.01	0.01	0.01	0.01	0.01	0.01
Premix^3^	0.20	0.20	0.20	0.20	0.20	0.20	0.20	0.20
Determined analysis								
Dry matter	89.3	90.1	89.8	89.9	89.7	89.9	89.7	90.6
Gross energy (kcal/kg)	3,476	3,510	3,576	3,622	3,661	3,678	3,709	3,759
Crude protein	15.2	15.6	16.4	16.9	15.3	15.5	16.6	17.2
Amino acid								
Arg	1.02	1.09	1.13	1.19	1.04	1.09	1.15	1.23
Lys	0.77	0.84	0.86	0.92	0.79	0.83	0.88	0.95
Met	0.45	0.48	0.53	0.54	0.45	0.48	0.50	0.52
Met + Cys	0.68	0.75	0.80	0.84	0.70	0.75	0.77	0.84
Thr	0.58	0.60	0.65	0.67	0.60	0.63	0.65	0.70
Trp	0.19	0.21	0.21	0.22	0.18	0.21	0.22	0.23
Ile	0.67	0.69	0.73	0.77	0.64	0.69	0.71	0.79
Val	0.74	0.76	0.81	0.83	0.71	0.78	0.82	0.87
Total ash	12.5	13.0	13.0	12.0	12.9	12.2	12.2	13.1
Feed particle size								
GMD^4^ (µm)	854	912	864	943	983	942	1,025	987
GSD^5^ (µm)	2.54	2.48	2.47	2.37	2.22	2.25	2.16	2.15
Calculated analysis^6^								
AMEn (kcal/kg)	2,680	2,680	2,680	2,680	2,780	2,780	2,780	2,780
Ether extract	3.37	3.58	3.79	4.00	5.03	5.26	5.49	5.72
Crude fiber	3.70	3.70	3.70	3.70	3.70	3.71	3.71	3.72
SID AA^7^								
Arg	0.92	0.97	1.02	1.08	0.92	0.97	1.02	1.08
Lys	0.68	0.72	0.76	0.80	0.68	0.72	0.76	0.80
Met	0.40	0.43	0.46	0.49	0.40	0.43	0.46	0.49
Met + Cys	0.62	0.65	0.69	0.73	0.62	0.65	0.69	0.72
Thr	0.50	0.53	0.56	0.58	0.50	0.53	0.56	0.59
Trp	0.16	0.17	0.18	0.19	0.16	0.17	0.18	0.19
Ile	0.57	0.60	0.63	0.66	0.57	0.60	0.63	0.66
Val	0.64	0.68	0.71	0.74	0.64	0.68	0.71	0.74
Calcium	3.83	3.83	3.83	3.83	3.90	3.90	3.90	3.90
Available phosphorus	0.37	0.37	0.37	0.37	0.38	0.38	0.38	0.38

1 Standardized ileal digestible lysine

2 70% coarse (3 to 4 mm) and 30% fine (<0.6 mm)

3 Provided the following per kilogram of diet: vitamin A (*trans*-retinyl acetate), 8,000 IU; vitamin D_3_ (cholecalciferol), 3,000 IU; vitamin E (dl-α-tocopheryl acetate), 10 IU; vitamin K, 1 mg; vitamin B_1_, 1.0 mg; vitamin B_2_, 4 mg; vitamin B_6_, 1.5 mg; vitamin B_12_ (cyanocobalamin), 10 mg; niacin, 20 mg; pantothenic acid (d-calcium pantothenate), 8.2 mg; folic acid, 1 mg; biotin, 100 μg; choline (choline chloride), 200 mg; manganese (MnO), 70 mg; zinc (ZnO), 50 mg; iron (FeSO_4_.H_2_O), 30 mg; copper (CuSO_4_ 5H_2_O), 6 mg; iodine [Ca(IO_3_)2], 0.5 mg; selenium (Na_2_SeO_3_), 0.3 mg; Axtra PHY, 30mg [300 U of 4a24 6-phytase (EC 3.1.3.26)] supplied by DuPont, Madrid, Spain.

4 Geometric mean diameter.

5 Log normal standard deviation.

6 According to FEDNA (2019).

7 Standardized ileal digestible amino acid.

### Laboratory Analysis

Particle size distribution and mean particle size of the diets, expressed as geometric mean diameter (**GMD**) ± geometric standard deviation (**GSD**), were determined in 100 g samples using a shaker (Retsch, Stuttgart, Germany) provided with eight sieves ranging in mesh from 5,000 to 40 μm as outlined by ASAE (2003). Representative samples of the diets were ground in a laboratory mill (Retsch Model Z-I, Stuttgart, Germany) equipped with a 0.75-mm screen and analyzed for moisture by oven-drying (method 930.15), total ash using a muffle furnace (method 942.05), and nitrogen by combustion (method 968.06) using a Leco analyzer (model FP-528, Leco Corp., St. Joseph, MI) as indicated by AOAC International (2005). The gross energy of the diets was determined in an adiabatic bomb calorimeter (model 6400, Parr Instrument Company, Moline, IL) and the AA composition was analyzed by ion-exchange chromatography (Hewlett-Packard 1100, Waldbronn, Germany) as described by De Coca-Sinova et al. (2008). For the determination of methionine and cysteine, separate feed samples were oxidized with performic acid before hydrolysis and measured as Met sulfone and cysteic acid, respectively. Tryptophan was determined after alkaline hydrolysis for 20 h at 110 ºC. All the analyses were conducted in duplicate except for the GMD ± GSD that was determined in triplicate.

### Measurements

#### Egg Production

All eggs produced were collected daily. Egg weight (**EW**) was measured in all eggs laid the first day of each week of the 10 experimental periods (4 wk each). The average EW by cage and period was used for further analyses. Feed disappearance and hen BW were determined by replicate by period and cumulatively. From these data, egg production, EW, egg mass, ADFI, feed conversion ratio per kilogram of eggs (**FCR**), and BW gain were determined by period and cumulatively (19−59 wk of age). In addition, energy intake, expressed as kcal AMEn ingested per hen per day, and energy efficiency, expressed as kcal/g of egg, were calculated. Any mortality was recorded and weighed as it occurred.

#### Egg Quality

The percentage of unsaleable eggs (dirty, broken, and shell-less) was determined in all eggs produced by two independent observers blind to treatment. An egg was considered as dirty when a spot of any kind or size was detected on the shell (Lázaro et al., 2003). Egg shell strength and HU were measured in eight fresh eggs collected randomly from each replicate for the last 2 d of each of the ten experimental periods. Eggs were individually weighed and the strength of the shell, expressed in g/cm^2^, was determined applying increased pressure to the broad pole of the egg, using an egg shell force gauge (Egg Force Reader, SANOVO Technology A/S, Odense, Denmark) as indicated by Safaa et al. (2008a). Haugh Units were measured using a multi tester equipment (QCM System, Technical Services and Supplies, Dunnington, York, UK) as indicated by Pérez-Bonilla et al. (2012a). The proportion of egg components (% of egg weight) was measured in ten eggs produced in the last 2 d of the last two experimental periods, exclusively. The yolk and the shell with the membranes, were separated and weighed as indicated by Grobas et al. (2001). Albumen weight was estimated by difference between the weight of the egg and the weight of the yolk plus the shell, as recommended by Safaa et al. (2008b).

### Statistical Analysis

Data were analyzed as a completely randomized design with eight treatments arranged as a 2 × 4 factorial, with AMEn and DLys content of the diets as main effects, using the MIXED procedure of SAS (SAS Institute, 2004). Each treatment was replicated nine times and the experimental unit was an enriched cage with nine hens for all measurements. When the effects of AMEn concentration and DLys content on the different variables studied were significant, average means were separated using the Tukey test. In addition, the effects of the level of DLys were partitioned into its linear (**L**) and quadratic (**Q**) components. The data were analyzed using the regression procedure (SAS Institute, 2004). The effects of age and the interaction between age of the hens and diet (AMEn and DLys content) on production and egg quality traits, were tested as indicated by Littell et al. (1998). Mortality values did not follow a normal distribution and consequently, the number of dead birds was analyzed as a binomial distribution, using the LOGISTIC procedure of SAS (SAS Institute, 2004). Results in tables are presented as means and the differences were considered significant at *P* < 0.05.

## RESULTS

The chemical analyses of the diets were in reasonable accordance with the calculated values. Health status of the birds was good and the mortality observed (6.4% as an average) was within the values expected for hens kept under commercial conditions.

### Hen Production and Egg Quality

No interactions AMEn and DLys content of the diets were observed for any of the production and egg quality traits studied. Consequently, only main effects are presented (Tables 2 and 3, respectively). Age affected (*P* < 0.001) all egg production and egg quality traits studied except mortality and proportion of egg components, but no interactions between age of the hens and diet were detected.

**Table 2:** Influence of energy (AMEn/kg) and standardized ileal digestible lys (DLys) contents of the diet on egg production from 19 to 59 wk of age.

	AMEn (kcal/kg)		DLys (%)					*P*-value^2^			
	2,680	2,780	0.68	0.72	0.76	0.80	SEM (n = 9)^1^	Main effects^3^		Regression^4^	
								AMEn	DLys	L	Q
DLys intake (mg/d)	819	811	744^d^	793^c^	843^b^	883^a^	7.24	0.157	<0.001	<0.001	0.417
Energy intake^5^	297^b^	305^a^	299	301	303	301	2.56	0.032	0.121	0.253	0.433
Egg rate (%)	85.9	86.3	84.8	86.4	86.7	86.5	1.63	0.747	0.620	0.252	0.390
Feed intake (g/d)	110.7	109.6	109.4	110.1	110.9	110.4	0.95	0.127	0.437	0.188	0.384
Egg weight (g)	60.2^b^	61.3^a^	60.1^b^	60.5^b^	61.0^ab^	61.4^a^	0.41	<0.001	0.013	0.003	0.816
Egg mass (g/d)	51.7	52.9	51.0	52.2	52.9	53.1	1.03	0.119	0.157	0.036	0.506
FCR^6^ (kg/kg)	2.148^a^	2.079^b^	2.148	2.113	2.106	2.086	0.0410	0.017	0.458	0.144	0.797
BW^7^ (g)	1,763	1,769	1,762	1,770	1,768	1,764	17.8	0.702	0.625	0.262	0.997
Mortality^8^	0.0581	0.0732	0.0455	0.0707	0.0707	0.0758		0.394	0.628	0.501	0.519
Energy efficiency^9^	5.68	5.70	5.79	5.69	5.66	5.61	0.11	0.735	0.441	0.124	0.802

^a-d^Values with different superscript letters are significantly different (*P* < 0.05).

1 Standard error of the mean (36 and 18 replicates for the AMEn and DLys effects, respectively).

2 Age effect was significant for all the variables studied (*P* < 0.001) except for bird mortality.

3 The interactions were not significant for any of the variables studied (*P* > 0.10).

4 The effects of the level of DLys on the different variable studied were partitioned into its linear (L) and quadratic (Q) components.

5 Kcal AMEn/day.

6 Feed conversion ratio.

7 BW determined at 59 wk of age.

8 Expressed as the proportion of dead birds with respect to total number of birds per cage.

9 Kcal of AMEn/g of egg.

**Table 3:** Influence of energy (AMEn/kg) and standardized ileal digestible lys (DLys) contents of the diet on egg quality traits from 19 to 59 wk of age.

	AMEn (kcal/kg)		DLys (%)					*P*-value^2^			
	2,680	2,780	0.68	0.72	0.76	0.80	SEM (n = 9)^1^	Main effects^3^		Regression^4^	
								AMEn	DLys	L	Q
Egg quality											
Haugh units	95.6	95.2	95.6	95.6	95.8	96.0	0.34	0.257	0.459	0.128	0.255
Shell strength (g/cm^2^)	4,542	4,572	4,519	4,571	4,585	4,553	0.0658	0.457	0.855	0.598	0.570
Unsaleable eggs^5^ (%)	2.23	1.92	2.16	2.31	1.96	1.88	0.259	0.357	0.506	0.190	0.478
Dirty (%)	0.97	0.88	0.98	0.95	0.86	0.91	0.137	0.230	0.773	0.489	0.862
Broken (%)	0.84	0.75	0.73	0.94	0.77	0.73	0.222	0.510	0.576	0.658	0.290
Shell-less (%)	0.42	0.30	0.45	0.42	0.32	0.25	0.106	0.364	0.466	0.038	0.746
Egg components^6^ (% of the egg)											
Albumen	60.4	60.4	60.3	60.3	60.5	60.4	0.25	0.813	0.774	0.451	0.709
Yolk	26.3	26.4	26.3	26.4	26.3	26.3	0.19	0.524	0.987	0.984	0.948
Shell	13.3	13.2	13.4	13.4	13.2	13.3	0.18	0.281	0.593	0.274	0.508

1 Standard error of the mean (36 and 18 replicates for the AMEn and DLys effects, respectively).

2 Age effect was significant for all the variables studied (*P* < 0.001) except for the proportion of egg components.

3 The interactions were not significant for any of the variables studied (*P* > 0.10).

4 The effects of the level of DLys on the different variable studied were partitioned into its linear (L) and quadratic (Q) components.

5 ∑ Dirty, broken, and shell-less eggs.

6 Determined in the last two experimental periods (52 to 59 wk of age), exclusively. Shell percentage was estimated as the difference between egg weight and albumen and yolk weights.

#### Energy Concentration of the Diet

**Figure 1:**
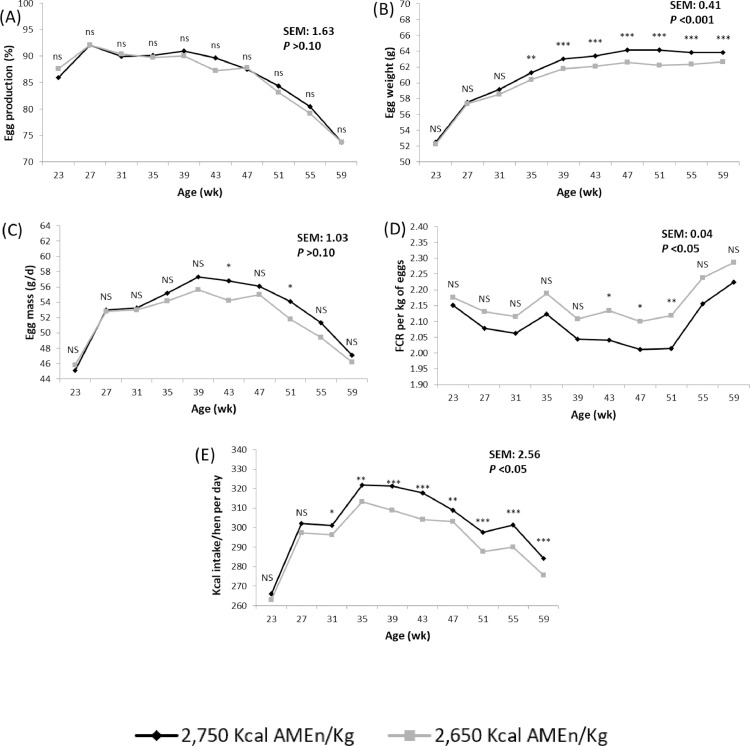
Influence of the energy content (AMEn/kg) of the diet on egg production (A), egg weight (B), egg mass (C), feed conversion ratio (D), and kcal of energy intake (E) from 19 to 59 wk of age^1^. ^NS^*P* > 0.05; *0.05 > *P* > 0.01; ^⁎⁎^0.01 > *P* > 0.001; ^⁎⁎⁎^*P* < 0.001. ^1^Age effect (*P* < 0.001).

An increase in the AMEn content of the diet from 2,680 to 2,780 kcal/kg improved EW (60.2 vs. 61.3 g; *P* < 0.001), FCR (2.148 vs. 2.079; *P* < 0.05), and energy intake (296.7 vs. 304.7 kcal; *P* < 0.05) but had no effect in any of the other variables studied. Most of the differences observed were detected after the 3^rd^ period of the laying cycle for EW and energy intake, and after the 5^th^ period for FCR (Figure 1). Energy content of the diet did not affect the incidence of dirty, broken, or shell-less eggs, or the proportion of yolk, albumen, and shell of the eggs.

#### Digestible Lysine Content

**Figure 2:**
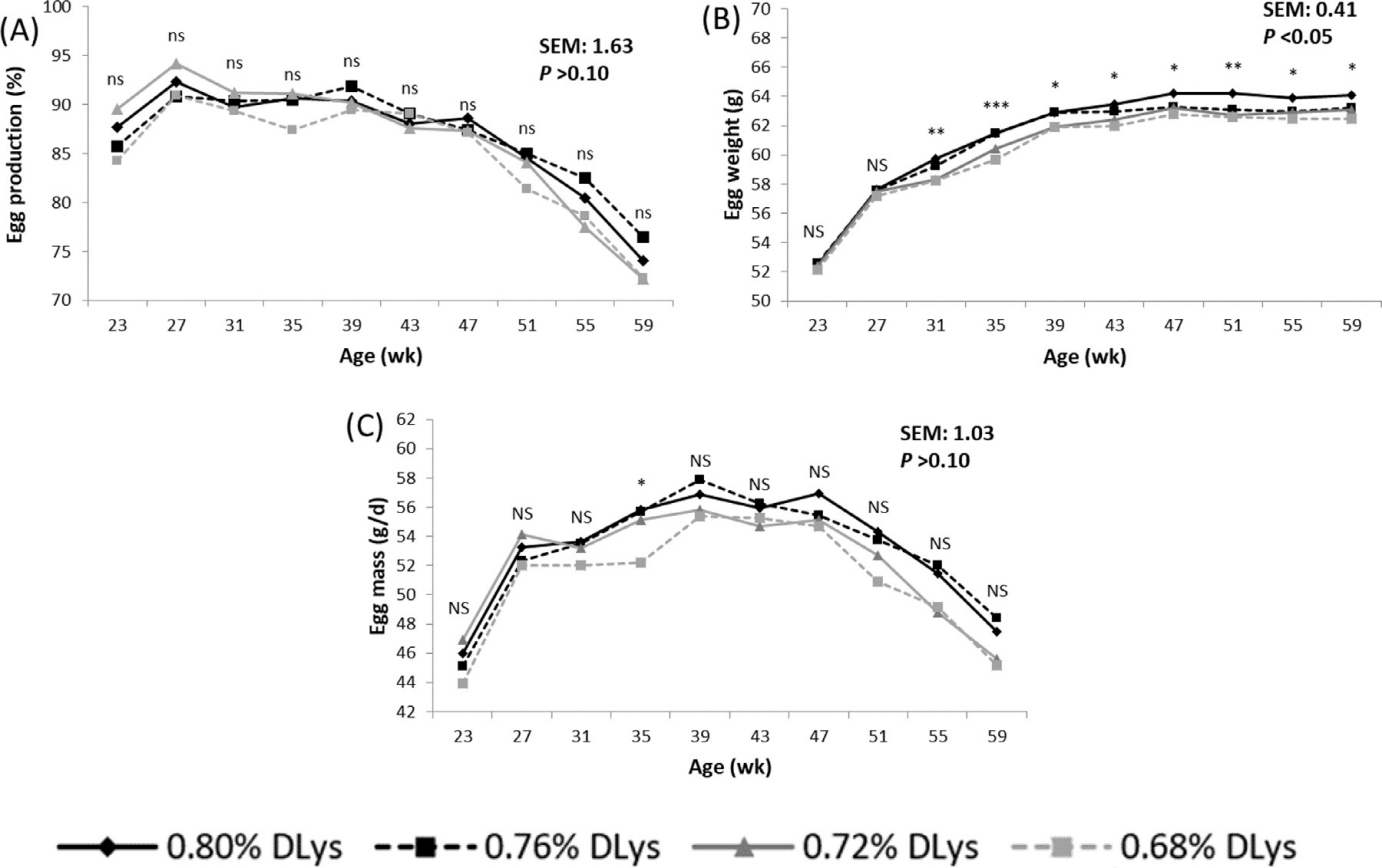
Influence of the standardized ileal digestible lysine (DLys) content of the diet (%) on egg production (A), egg weight (B) and egg mass (C) from 19 to 59 wk of age^1^. ^NS^*P* > 0.05; *0.05 > *P* > 0.01; ^⁎⁎^0.01 > *P* > 0.001; ^⁎⁎⁎^*P* < 0.001. ^1^Age effect (*P* < 0.001).

An increase in the DLys content of the diet from 0.68% to 0.80% did not affect egg production, FI, BW, energy intake, or FCR in any of the periods considered. However, EW (L, *P* < 0.01) and egg mass production (L, *P* < 0.05) increased as the level of DLys increased (Table 2). Most of the effects of DLys on EW were observed after the first two periods of the egg laying cycle, once egg production was above 90% (Figure 2). Diet did not affect any of the egg quality traits studied, except the percentage of shell-less eggs that decreased as the level of DLys increased (L, *P* < 0.05). The proportion of egg components, measured only in the last two experimental periods, was not affected by the DLys content of the diet.

## DISCUSSION

### Energy Content of the Diet

The energy (and nutrient) concentration of the diet did not affect egg production, in agreement with previous reports (Leeson et al., 2001; Jalal et al., 2006; Wu et al., 2007; Saldaña et al., 2016). An increase in energy from 2,680 to 2,780 kcal/kg (a 3.8% increase) decreased ADFI by 1.0% and increased egg mass by 2.3%, although the differences were not significant (*P* = 0.13 and *P* = 0.12, respectively). Perez-Bonilla (2012a) reported a 4.4% decrease in ADFI with an increase in egg mass of 3.5% as the AMEn of the diet increased from 2,650 to 2,950 kcal/kg, consistent with the data reported herein. Guzmán et al. (2016) however, did not find any increase in egg mass production when the AMEn of the diet increased from 2,650 to 2,750 kcal/kg. The reason for the discrepancy among authors on the effects of energy concentration of the diet on egg mass production is not known but might depend on factors such as the ingredient composition and nutrient content of the control diet (i.e., energy content and level of supplemental fat), management and environmental conditions (i.e., humidity and temperature of the barn), and strain and age of the hens (i.e., BW, egg rate, and EW).

In the current research, EW increased as the AMEn content of the diet increased, consistent with data of Grobas et al. (1999b), Harms et al. (2000), Wu et al. (2005), and de Persio et al. (2015). However, Mathlouthi et al. (2002) and Valkonen et al. (2008) did not find any change in EW with increases in the energy content of the diet of up to 300 kcal/kg. The reasons for the discrepancy among authors on the effects of the energy concentration of the diet on EW are not apparent. Under most practical conditions, an increase in dietary energy is accompanied by an increase in the level of LNL and supplemental fat (Grobas et al., 2001; Pérez-Bonilla et al., 2012a). In the current research, the level of LNL and supplemental fat of the experimental diets increased from 1.8% to 2.9% and from 1.3% to 3.5%, respectively, as the energy content increased. The requirements in LNL of the hens to maximize egg size are a subject of debate. Scragg et al. (1987) reported a linear increase in EW as the LNL content of the diet increased from 0.79% to 2.33%. However, Jensen et al. (1958), Whitehead (1981), and Grobas et al. (1999a,c) reported that LNL levels as low as 0.9% to 1.1% were sufficient to maximize EW, and that once this minimum level was achieved, the level of supplemental fat was responsible for increase in EW observed. In fact, Grobas et al. (1999b, 2001), Safaa et al. (2008b), and Herrera et al. (2017, 2018) reported that the increase in EW observed with increases in the level of supplemental fat was independent of the LNL content of the diet. Moreover, Bouvarel et al. (2010) estimated that EW increased by 0.2 g per each 1% increase in supplemental fat. The information provided confirmed that the increase in EW observed when high energy diets are used should be attributed primarily to an increase in supplemental fat rather than to an increase in LNL content.

An increase in the AMEn of the diet from 2,680 to 2,780 kcal (a 3.8% increase) increased energy intake by 2.7% and improved FCR by 3.2%, in agreement with most published research (Grobas et al., 1999a; Wu et al., 2005; Pérez-Bonilla et al., 2012a; and Saldaña et al., 2016). The data suggest that laying hens do not regulate accurately FI according to their energy requirements and tend to over consume energy as the AMEn of the diet increases (Bouvarel et al., 2011; dePersio et al., 2015; Mateos et al., 2019). The supplemental fat used in the current research was 2.2% greater for the high than for the low energy diet. The efficiency of AME to net energy is higher for lipid sources than for carbohydrates sources and consequently, an increase in dietary fat improves energy efficiency, especially in high producing laying hens. Moreover, supplemental fat reduces transit time through the gastrointestinal tract of the bird, facilitating the contact between digesta and enzymes and the utilization of other dietary components (Mateos and Sell, 1980, 1981a). In fact, Mateos and Sell (1981b,c) reported that the digestibility of simple sugars (i.e., sucrose) increased when the level of supplemental fat of the diet increased from 0% to 7%. In addition, supplemental fat agglomerates the fines present in the diet, increasing palatability and feed intake of the hens (Bouvarel et al., 2011; Mateos et al. 2012, 2019).

The influence of the energy content of the diet on egg quality traits was of limited practical interest, in agreement with data of Grobas et al (1999a), Valkonen et al. (2008), and Saldaña et al. (2016) for HU, Jiang et al. (2013), Valkonen et al. (2008), and Saldaña et al. (2016) for shell quality, and Grobas et al. (1999b), dePersio et al. (2015), and Saldaña et al. (2016) for egg components.

### Digestible Lysine Content

An increase in the DLys content of the diet from 0.68 to 0.80% (744 to 883 mg DLys/d) did not affect FI or egg production, consistent with data of Schutte and Smink (1998) in white hens fed diets varying in apparent faecal digestible Lys (**AFLys**) from 0.49 to 0.77% (539 to 847 mg AFLys/d). Wijtten et al. (2006) reported that in the peak production phase, brown hens require 650 mg AFLys/d to maximize egg production, a recommendation below the lowest value of the DLys range used in the current research. More recently, Rocha et al. (2009) and Kumar et al. (2018) reported that white hens require 759 and 769 mg of digestible Lys/d, respectively, to maximize egg production. The data reported herein indicate that the lower amount of Lys used in the current research (744 mg DLys/d, corresponding to 0.68% of the diet) was sufficient to maintain an adequate level of egg production. This recommendation agrees with most research published in recent years but is higher than the 593 mg DLys/d recommended by the NRC (1994).

In the current research, EW (and egg mass production) increased linearly as the DLys content of the diet increased from 0.68 to 0.80% (744 to 883 mg DLys/d), in agreement with data of Novak et al. (2004) and Schmidt et al. (2008) in white hens in the first and second cycle of egg production, respectively. Similar values (831 and 855 mg DLys/d) have been reported by Lemme (2009) and Van Krimpen et al. (2015) based in the data of two meta-analytical studies which included 19 and six trials (mixed strain of hens), respectively. Kumar et al. (2018) reported that the DLys intake needed to optimize hen production varied depending on the trait studied. Values reported by these authors in mg/d, were 769 for egg production, 903 for EW, and 839 for feed efficiency, data that are consistent with the results reported herein. Coon and Zhang (1999), however, reported that 705 mg DLys/d were sufficient to maximize egg mass production in white hens in the peak of egg production, a recommendation below the lowest value of the range used in the current research. Similarly, Wijtten et al. (2006) reported that egg production of brown hens increased linearly as the AFLys content of the diet increased from 0.49 to 0.67%, suggesting that hens required at least 700 mg/d for optimal egg mass production. Schmidt et al. (2008) and Rocha et al. (2009) reported increases in EW as the DLys content of the diet increased from 0.55% to 0.77%, suggesting that white hens required at least 788 and 759 mg DLys/d, respectively for maximizing EW. Similarly, Kakhki et al. (2016) observed, also in white hens, that EW (and egg mass production) increased as the DLys content of the diet increased from 0.66 to 0.81%, suggesting a minimum requirement of at least 778 mg Dlys/d to maximize EW. In the current research, FCR and energy efficiency improved from 2.15 to 2.09 and from 5.79 to 5.61, respectively, as the level of DLys increased from 0.68 to 0.80%, in agreement with data of Novak et al. (2004). However, in our experiment the difference observed did not reach significance (L; *P* = 0.14 and *P* = 0.12, respectively). Kumar et al. (2018) observed also that the requirements for DLys were lower for feed efficiency than for EW and higher for both variables than for egg rate.

The DLys content of the diet and the DLys intake of the hens had limited effects on egg quality, in agreement with data of Bouvarel et al. (2010) and Spangler et al. (2018). In fact, the only trait affected was the incidence of shell-less eggs that decreased as the level of DLys of the diet increased. We do not have any clear explanation on the observed benefits of DLys on this trait. Kakhki et al. (2016) reported an increase in HU as the DLys content of the diet increased from 0.66% to 0.87%. Also, Kumar et al. (2018) observed that the incidence of cracked eggs increased linearly as the DLys content of the diet increased. The proportion of egg components was not affected by the DLys intake of the hens, in agreement with of Rocha et al. (2009), and Spangler et al. (2018) in white hens. Novak et al. (2004), however, reported a significant increase in the proportion of albumen as the dietary Lys increased from 44 to 63 wks of age but not from 20 to 43 wks of age. Whitehead et al. (1991), Bouvarel et al. (2010), and Kakhki et al. (2016) indicated that the proportion of egg components depends primarily of age, with nutrient factors being of low relative interest. Pastore et al. (2018) suggested that Lys is used primarily to support egg production and only when in excess, this AA is used to modify the synthesis of the egg components, consistent with the results reported herein.

In summary, an increase in the energy content of the diet from 2,680 to 2,780 kcal AMEn/kg, did not affect egg production but increased egg weight, probably because of the higher level of supplemental fat of the high energy diets. Brown hens require no more than 744 mg DLys per day (corresponding to 0.68% DLys in the diet) to optimize egg production. However, when the objective is to maximize egg weight (and egg mass production), brown-egg laying hens should consume at least 843 mg DLys per day. The data confirm that the digestible Lys requirements of laying hens depend on the response criteria studied, being at least 100 mg/d greater for maximizing egg weight than for optimizing the number of eggs produced.

## DISCLOSURES

The authors confirm that there are not conflicts of interest in this research.
